# Differential response to intravitreal dexamethasone implant in naïve and previously treated diabetic macular edema eyes

**DOI:** 10.1186/s12886-020-01716-2

**Published:** 2020-11-11

**Authors:** Javier Zarranz-Ventura, Barbara Romero-Núñez, Carolina Bernal-Morales, Daniel Velazquez-Villoria, Anna Sala-Puigdollers, Marc Figueras-Roca, Sergio Copete, Laura Distefano, Anna Boixadera, Jose García-Arumi, Alfredo Adan, on behalf of the Hospital Clínic - Hospital Vall de Hebron Intravitreal Dexamethasone Implant study group, Alfredo Adan, Socorro Alforja, Carolina Bernal-Morales, Anna Boixadera, Ricardo P. Casaroli-Marano, Sergio Copete, Laura Distefano, Marc Figueras-Roca, Jose García-Arumi, Joan Giralt, Victor Llorens, Marina Mesquida, Manuel J. Navarro-Angulo, Laura Pelegrin, Barbara Romero-Núñez, Anna Sala-Puigdollers, Daniel Velazquez-Villoria, Miguel Angel Zapata, Javier Zarranz-Ventura

**Affiliations:** 1 Institut Clínic d’Oftalmología (ICOF), Hospital Clinic, C/ Sabino Arana 1, 08028 Barcelona, Spain; 2 Institut de Investigacions Biomediques August Pi i Sunyer (IDIBAPS), Barcelona, Spain; 3 Departmento de Oftalmología, Hospital Vall de Hebron, Barcelona, Spain

**Keywords:** Diabetic macular edema, Real world setting, Naïve, Previously treated, Refractory, Dexamethasone, Implant, Ozurdex, Audit, Benchmark standard

## Abstract

**Background:**

To identify different response patterns to intravitreal dexamethasone implants (IDI) in naïve and previously treated (PT) diabetic macular edema (DME) eyes in a real-life setting.

**Methods:**

342 IDI injections (203 DME eyes) were included. Number of IDI injections, percentage (%) of eyes with 1, 2, 3 and ≥ 4 injections, time to reinjections, visual acuity (VA), intraocular pressure (IOP) and central retinal thickness (CRT) were evaluated for naïve and PT DME eyes over 24 months.

**Results:**

Mean number of injections was significantly lower in naïve vs PT DME eyes (1.40 ± 0.9 vs 1.82 ± 0.9, *p* < 0.001). The percentage of eyes receiving 1 injection was significantly higher in naïve vs PT DME eyes (76.1 vs 47.7), (*p* < 0.001). However, it was significantly lower for 2 (16.4 vs 29.4), or 3 injections (1.4 vs 17.6) (both *p* < 0.001), with no differences in eyes receiving ≥4 injections (5.9 vs 5.1 respectively, *p* = 0.80). Mean time to reinjection was not significantly different between both groups for the second, third and fourth injection (9.6 ± 4.0 vs 10.0 ± 5.5, *p* = 0.75, 13.2 ± 4.0 vs 16.0 ± 3.5, *p* = 0.21 and 21.7 ± 3.8 vs 19.7 ± 5.8, *p* = 0.55). VA scores were consistently better in naïve vs PT DME eyes at all studied timepoints, with no significant differences in CRT reduction or adverse effect rates.

**Conclusion:**

Naïve DME eyes received lower number of IDI injections and showed better VA levels than PT DME eyes for 24 months in a real-world setting. This data supports the IDI use in early DME stages and provide further evidence of better IDI response when used as first-line therapy.

## Summary statement

Intravitreal dexamethasone implants provide better response in naïve than previously treated diabetic macular edema eyes with better visual outcomes and lower number of injections over 24-months, in a large real-world cohort of eyes treated in routine clinical care outside clinical trial criteria.

## Background

Diabetic macular edema (DME) is a complex disease of multifactorial origin in which fluid accumulates in the retinal layers due to the disruption of blood retinal barrier and increased vascular permeability [1–3]. Treatment options to manage DME include intravitreal injections of anti-vascular endothelial growth factor (anti-VEGF) or intravitreal corticosteroids (i.e. dexamethasone, fluocinolone acetonide and triamcinolone acetonide) [4–6]. The intravitreal dexamethasone implant (IDI, 0.7 mg, Ozurdex®: Allergan, Inc., CA, USA) is a biodegradable, sustained-release drug delivery system that releases dexamethasone into the vitreous for up to 6 months, and is currently approved for the treatment of macular edema secondary to retinal vein occlusion [7], non-infectious posterior uveitis [8] and DME, based on the results of the MEAD trial [9]. This study pooled the data from 2 randomized, multicenter, masked, sham-controlled, phase 3 clinical trials (﻿ClinicalTrials.gov identifiers NCT00168337 and NCT00168389), that demonstrated visual and anatomic improvements in DME eyes, which were confirmed in subsequent trials [10, 11].

However, significant concerns appear with regards to the applicability of clinical trials results to real world scenarios. First, in clinical practice, the selection criteria are less strict, usually limited to failure of other therapies. This fact is especially relevant as eyes treated with IDIs in routine clinical practice are often those in which other therapies have primarily failed (laser, anti-VEGFs, etc.) and are at risk of developing chronic macular edema, limiting their potential for greater visual gains. Second, the visit and treatment schedule applied in clinical trials rarely reflects real world clinical conditions. In particular, in the MEAD trial reinjections of the IDI could not be performed prior to 6-months, limiting the potential of the implant to achieve greater visual gains in poor responsive eyes. Third, the potential loss of follow-up visits can produce an overestimation of the benefits and/or an underestimation of its side effects, affecting either way the outcomes reported in comparison to clinical trials. For these different reasons, it is important to evaluate the IDI performance in real-life scenarios.

Currently, anti-VEGF therapies are considered the first-line therapy for DME, and meanwhile the importance of corticosteroid therapy has been recognized it is mainly employed as a second-line therapy. As suggested by the EURETINA guidelines for the management of DME [12], IDIs are only considered as first-line therapy in patients whose medical history excludes the use of anti-VEGF therapies or in specific conditions: history of major cardiovascular events, unwillingness to receive monthly injections, or pseudophakic patients. Nevertheless, there is a growing body of evidence in the last years supporting the benefits of IDIs in naïve DME patients, and several studies have reported better visual outcomes compared to refractory DME eyes [13, 14]. In contrast, very few studies have evaluated specifically the treatment frequency and the number of injections in naïve DME eyes compared to previously treated eyes.

Thus, the purpose of the present study is to audit the use of the IDI in a large series of DME eyes treated in real-life clinical conditions, to identify different treatment patterns in naïve versus previously treated eyes. The study was performed over a 5-year period at two tertiary referral retinal units from a well-defined geographic area that covered a population of 1.8 million individuals. In addition, a specific sub-analysis was carried out to identify differences in baseline characteristics, VA, anatomical outcomes, number of injections and reinjection frequency in naïve eyes (defined as eyes with no prior intravitreal therapies) and previously treated eyes (which previously received intravitreal drugs). The results obtained were compared to those reported in the literature in clinical trials (e.g., MEAD, CHAMPLAIN, BEVORDEX) and previous real world published studies, to address the performance of IDI in a large cohort of unselected DME eyes in real-life conditions.

## Methods

### Study design

This study was approved by the Institutional Review Board (IRB) at the Hospital Clinic of Barcelona and it was conducted in accordance with the Tenets set forth in the Declaration of Helsinki (HCB/2016/0905). Clinical data were collected retrospectively from 2 specialized tertiary referral retina clinics in Barcelona (Spain): Institut Clínic de Oftalmología (ICOF) at Hospital Clinic of Barcelona and Hospital Vall d’Hebrón. No written informed consent was required as data was retrospectively collected from routine clinical care, as approved by the reference IRB. All eyes receiving IDI injections for DME between October 2010 and May 2015 were included in the study. A comprehensive dataset was distributed and completed in both study centers within the predetermined timeframe. Patient identifiers were removed to anonymize the data, and data from the individual centres were collated and merged into a centralized database for analysis.

### Clinical data collection

Data collected included demographics (age, gender, etc.), laterality, previous local treatments, number of previous injections, number of injections, surgical details, complications, current topical treatments, visual acuity (VA), intraocular pressure (IOP) and central retinal thickness (CRT) assessed by optical coherence tomography (OCT). This data was collected at all the study timepoints: baseline, 1–2 weeks, 6–8 weeks, and 3, 6, 9, 12, 18 and 24-months post-injection of the first IDI. Additional data was collected at each individual repeated injection during the study, including VA, IOP and CRT data prior to the procedure, and 1–2 weeks, 6–8 weeks, and 3 and 6-months post-procedure.

### Data sources/outcome measurements

All original data were gathered in routine clinical care visits. All injections data (i.e. injection date, number of injections, pre and post-injection data) were collected as described above. At each time point, VA was determined as the best VA with habitual correction or pinhole, rather than as the best-corrected refracted VA and presented in logarithm of the mínimum angle of resolution (logMAR) units. The analysis of eyes with a low VA was undertaken by substituting counting fingers (CF) and hand movement (HM) with 2.0 and 2.3 logMAR, respectively [15]. IOP measurements were obtained by Goldmann tonometry and presented in mmHg. The CRT was determined by OCT imaging using one of 2 different devices depending on the participating center (Hospital Clínic, Cirrus HD-OCT**®**, Dublin, CA, USA and Hospital Vall de Hebrón, Spectralis OCT**®**, Heidelberg Engineering, Germany). No research softwares were employed to control for inter-device measurement differences in CRT. No missing value substitutions were performed in patients where data were not available for a particular visit or were lost during follow-up.

### Statistical analysis

Descriptive, frequency statistics and the chi-squared test were used to assess the qualitative variables. The normality of quantitative variables was examined in histograms, and inter-group differences where evaluated with an independent Student’s T-test and Mann-Whitney U-test, when appropriate. A paired t-test was used to compare pre- and post-treatment changes. For VA change analysis, VA values are converted and presented in ETDRS letters. The cumulative probability of IOP events occurring after IDI injection are presented as survival curves using the Kaplan Meier (KM) method [16]. High IOP was defined as an IOP greater than 21 mmHg, 25 mmHg or 35 mmHg, as described elsewhere [17]. The probability of IOP elevation was evaluated for naïve and previously treated DME eyes subgroups, and KM survival curves were compared with the log-rank test. A *p*-value ≤0.05 was considered significant.

## Results

### Baseline demographic and clinical characteristics of study cohort

A total of 203 DME eyes from 179 patients treated with IDIs were included in this study. The baseline characteristics of the patients and study eyes are disclosed in Table 1. In this cohort, 67 eyes (33%) were treatment-naïve, whereas 136 eyes (67%) had previously received intravitreal treatment for DME. Previous intravitreal treatments included intravitreal triamcinolone (IVTA) in 27 eyes (13.3%), anti-VEGF drugs in 84 eyes (41.3%), and both IVTA and anti-VEGF treatment in 25 eyes (12.3%). Overall, previous laser treatment was performed in 154 eyes (75.8%), distributed as macular focal/grid laser therapy in 113 eyes (55.6%) and pan-retinal photocoagulation (PRP) in 110 eyes (54.1%). Mean baseline VA of the overall cohort was 0.92 logMAR (equivalent to 39 ETDRS letters) and mean CRT was 498.7 μm. A detailed comparison between naive and previously treated eyes at baseline is shown in Table 1.

**Table 1:** Patient demographics and clinical characteristics of the study eyes at baseline

Characteristic	Total (*N* = 203)	Treatment-naïve eyes (*N* = 67)	Previously treated eyes (*N* = 136)
Age mean years ± SD (range)	66.8 ± 10.3 (43–99)	66 ± 12.9 (43–99)	67 ± 8.9 (44–86)
Gender, n (%)			
Female	87 (42.9)	27 (40.3)	61 (44.9)
Male	116 (57.1)	40 (59.7)	75 (55.1)
Lens status in study eye, n (%)			
Phakic	105 (51.7%)	41 (61.2)	64 (47)
Pseudophakic	98 (48.3%)	26 (38.8)	72 (53)
Previous intravitreal therapy, n (%)			
IVTA	27 (13.3)	0 (0)	27 (19.8)
Anti VEGF	84 (41.3)	0 (0)	84 (61.7)
IVTA + Anti VEGF	25 (12.3)	0 (0)	25 (18.3)
Previous laser therapy, n (%)			
Any	154 (75.8)	38 (56.7)	116 (85.2)
Focal/grid	113 (55.6)	25 (37.3)	88 (64.7)
PRP	110 (54.1)	28 (41.7)	82 (60.2)
Mean VA, ETDRS letters (Snellen equivalent) ^a^	39 (20/160)	42.5 (20/160)	37.5 (20/200)
Mean CRT, μm (SD)	498.7 ± 136	482.1 ± 127.5	506.5 ± 139.7

*IVTA* Intravitreal triamcinolone acetonide, *VEGF* Vascular endothelial growth factor, *PRP* Pan-retinal photocoagulation, *VA* Visual acuity; ^a^Logarithm of the minimum angle of resolution values are converted into ETDRS letters, *CRT* Central retinal thickness

### Number of injections and treatment burden

In the overall cohort, a total of 342 IDI injections were administered in 203 eyes, with a mean number of injections of 1.68 ± 0.9 implants in a mean follow-up time of 16.3 ± 7.7 months (Table 2). The percentage of study eyes in the overall cohort receiving 1 injection was 57.1% (116 eyes), 2 injections was 25.1% (51 eyes), 3 injections was 12.3% (25 eyes) and > =4 injections was 5.4% (11 eyes). In this cohort, the mean time to reinjection was 9.95 ± 5.2 months for the 2nd injection, 15.71 ± 4.8 months for the 3rd injection and 20.4 ± 5.1 months for the 4th injection.

**Table 2:** Number of Injections and time to reinjection during the study period

	Total (*N* = 203)	Naïve (*N* = 67)	Previously treated (*N* = 136)	*P* value
Total number of Injections, N (%)	342 (100%)	94 (27.4%)	248 (72.6%)	
Number of Injections, Mean ± SD (Median-IQR)	1.68 ± 0.9 (1–1)	1.40 ± 0.9 (1–0)	1.82 ± 0.9 (2–1)	< 0.001
Follow up time (months), Mean ± SD (Median-IQR)	16.3 ± 7.7 (16.1–13.8)	14.5 ± 7.8 (12.4–13.7)	17.1 ± 7.6 (17.4–13.1)	0.02
Number of injections, n (% study eyes)				
1 injection	116 (57.1%)	51 (76.1%)	65 (47.7%)	< 0.001
2 injections	51 (25.1%)	11 (16.4%)	40 (29.4%)	0.04
3 injections	25 (12.3%)	1 (1.4%)	24 (17.6%)	< 0.001
≥ 4 injections	11 (5.4%)	4 (5.9%)	7 (5.1%)	0.80
Time to reinjection after 1st injection (months), Mean ± SD (Median-IQR)				
2nd injection	9.95 ± 5.2 (8–5)	9.61 ± 4.0 (8–3.75)	10.0 ± 5.5 (8–5)	0.75
3rd injection	15.71 ± 4.8 (15–7.5)	13.2 ± 4.0 (11–3)	16.0 ± 3.5 (16–7)	0.21
4th injection	20.4 ± 5.1 (19.5–8)	21.75 ± 3.8 (21.5–5.75)	19.75 ± 5.8 (18–5.25)	0.55

*SD* Standard deviation*, IQR* Interquartile range

When comparing naïve vs previously treated eyes, the mean number of injections was 1.4 ± 0.9 vs 1.82 ± 0.9 (*p* < 0.001) in a mean follow-up time of 14.5 ± 7.8 vs 17.1 ± 7.9 months (*p* = 0.02), respectively. The percentage of eyes receiving 1 injection was significantly higher in naïve vs previously treated eyes (76.1% vs 47.7%, *p* < 0.001), as was significantly lower in eyes requiring 2 injections (16.4% vs 29.4%, *p* = 0.04) or 3 injections (1.4% vs 17.6%, *p* = 0.001). No differences were observed in the percentage of eyes requiring > = 4 injections (5.9% vs 5.1%, *p* = 0.80). Interestingly, no differences between groups were observed in the time to reinjection for 2nd injection (9.61 ± 4.0 vs 10.0 ± 5.5 months, *p* = 0.75), 3rd injection (13.2 ± 4.0 vs 16.0 ± 3.5 months, *p* = 0.21) and 4th injection (21.75 ± 3.8 vs 19.75 ± 5.8 months, *p* = 0.55). All these results are presented in Table 2.

### Visual acuity outcomes

**Fig. 1:**
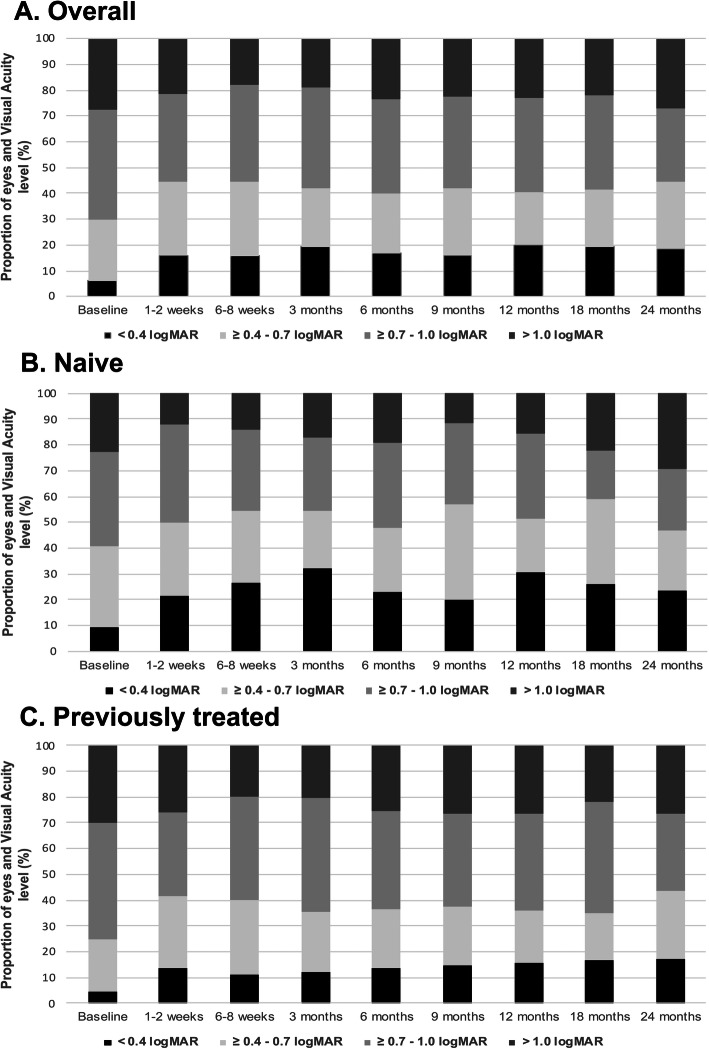
Distribution of diabetic macular edema (DME) study eyes by visual acuity level at all timepoints. **a**. Overall cohort. **b**: Naïve eyes. **c**: Previously treated eyes. At baseline, eyes with very good vision (black) and very poor vision (dark gray) were treated with the implant in routine clinical care. These two categories fall outside the MEAD clinical trial inclusion criteria and represent 33.5% of the study cohort (6 and 27.5%, respectively), highlighting the need for real world studies to evaluate the performance of the implant outside clinical trial scenarios. Naïve DME eyes showed consistently greater percentages of eyes with good vision (black and light gray) than previously treated DME eyes at all timepoints over 24 months. (Visual acuity levels; black: < 0.4 logMAR; light gray: ≥0.4–0.7 logMAR; medium gray: ≥0.7–1.0 logMAR; dark gray: > 1.0 logMAR)

The distribution of eyes by VA levels at each individual timepoints is presented in Fig. 1. In the overall cohort, at baseline the percentage of eyes with VA < 0.4 logMAR was 6%, VA ≥0.4–0.7 was 24%, VA ≥0.7–1.0 was 42.5% and VA > 1.0 was 27.5%. At 24 months, the percentage of eyes with VA < 0.4 logMAR was 18.5%, VA ≥0.4–0.7 was 25.7%, VA ≥0.7–1.0 was 28.5% and VA > 1.0 was 27.1%. The percentage of eyes with good VA levels (< 0.4 logMAR and ≥ 0.4–0.7 logMAR) was significantly higher in naïve vs previously treated eyes in all study timepoints (all *p* < 0.05), as presented in Fig. 1.

**Fig. 2:**
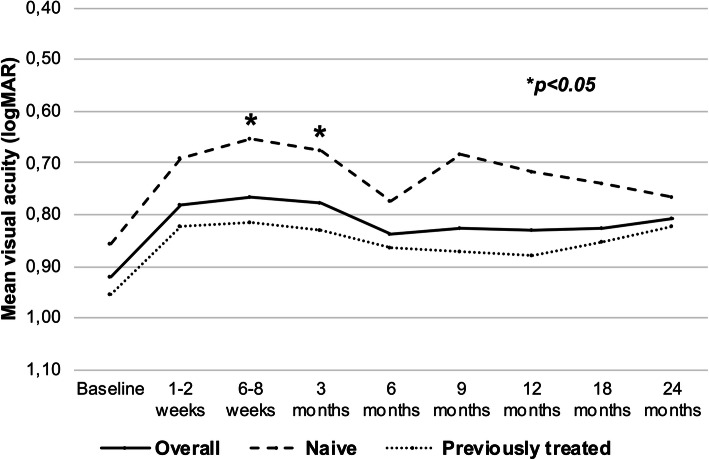
Visual acuity outcomes. Evolution of mean visual acuity from baseline to 24 months in the overall cohort (solid line), naïve eyes (stripped line) and previously treated eyes (dotted line). Significant differences were observed between naïve and previously treated eyes at 6–8 weeks and 3 months timepoints (*p < 0.05)

In the overall cohort, mean baseline VA was 0.92 ± 0.4 LogMAR, at 6–8 weeks was 0.76 ± 0.4 LogMAR and at 24 months was 0.8 ± 0.5 LogMAR (Fig. 2 and Table 3). At 24 months, mean VA improvement was + 6 letters at 24 months in the overall cohort, with no significant differences in subgroup analysis in treatment-naïve and previously treated eyes (+ 4.5 letters vs + 6.5 letters, *p* = 0.70, Table 3). However, treatment-naïve eyes maintained better mean VA at all timepoints compared to previously treated eyes, with significant differences at 6–8 weeks (mean VA 0.65 ± 0.47 vs. 0.81 ± 0,47, *p* < 0.05) and 3 months (mean VA 0.68 ± 0,53 vs. 0.83 ± 0.46, *p* < 0.05) (Fig. 2).

**Table 3:** Outcome measures before and 24 months after treatment in naïve vs previously treated eyes

	Total (*N* = 203)	Naïve (*N* = 67)	Previously treated (*N* = 136)	*p*-value
Mean VA, ETDRS letters				
Before treatment (mean ± SD) (Snellen equivalent)	39 ± 61 (20/160)	42.5 ± 57.5 (20/160)	37.5 ± 62.5 (20/200)	0.19
After treatment (mean ± SD)	45 ± 57.5 (20/125)	47 ± 60 (20/125)	44 ± 56 (20/125)	0.71
Mean Change	6	4.5	6.5	0.70
Mean CRT, μm				
Before treatment (mean ± SD)	498.7 ± 136	482.1 ± 127.5	506.5 ± 139.7	0.25
After treatment (mean ± SD)	402.6 ± 154.83	340.5 ± 88.28	427.4 ± 165	0.10
Mean Change	−96.1	−141.5	−79.0	0.46

*VA* Visual acuity*, ETDRS* Early treatment diabetic retinopathy study*, CRT* Central retinal thickness*, SD* Standard deviation*, IQR* Interquartile range

### Central retinal thickness outcomes

**Fig. 3:**
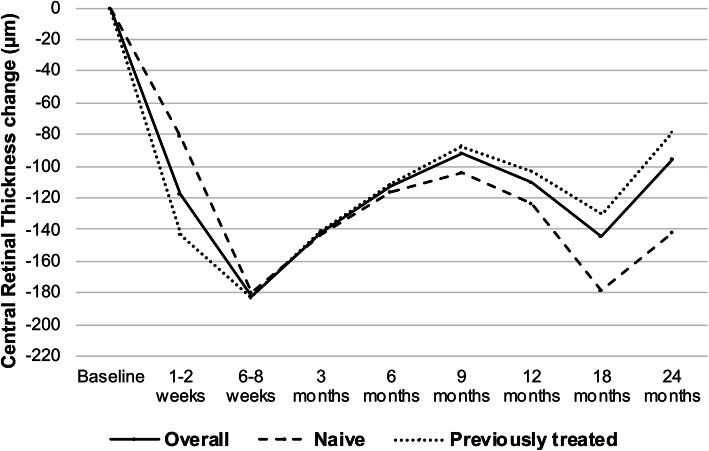
Anatomical outcomes. Evolution of mean central retinal thickness change from baseline to 24 months in the overall cohort (solid line), naïve eyes (stripped line) and previously treated eyes (dotted line). No significant differences were observed between naïve and previously treated eyes at any timepoint

The evolution of CRT changes from baseline in the overall, naïve and previously treated eyes cohorts is presented in Fig. 3 and Table 3. Significant improvements were observed in CRT at 6–8 weeks (− 181.8 μm, *p* < 0.05) and at 24 months (− 96.1 μm, *p* < 0.05). In the subgroup analysis, no differences were observed in CRT improvements between naïve and previously treated eyes at 6–8 weeks (− 179.7 μm vs − 182.8 μm) or 24 months (− 141.5 μm vs − 79.0 μm, *p* = 0.46).

### Intraocular pressure outcomes

**Fig. 4:**
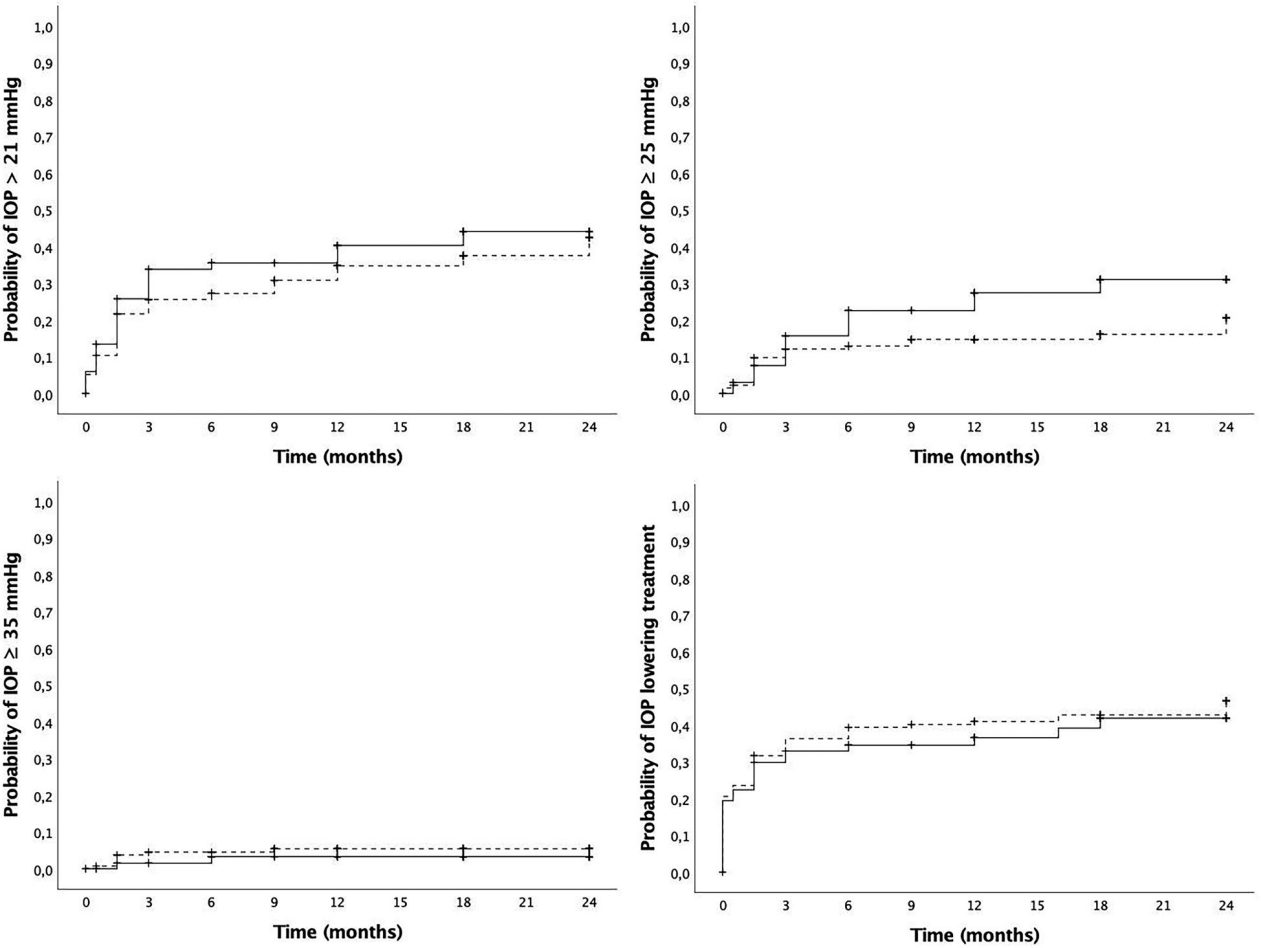
Intraocular pressure outcomes. Cumulative probability of different levels of intraocular pressure (IOP) elevations (top-left: > 21 mmHg, top-right: ≥25 mmHg, bottom-left: ≥35 mmHg) and IOP lowering treatment requirement (bottom-right). No significant differences were observed between naïve and previously treated eyes for any item at any timepoint

All IOP outcome measures are presented in Fig. 4. The cumulative probability of IOP ≥ 21 / 25 / 35 mmHg at 12 months was 50% / 23% / 6%, and at 24 months was 60% / 30% / 7%, respectively. No significant differences in IOP elevations were observed between naïve and previously treated eyes. At baseline, 41 eyes (20.2%) were already on treatment with topical IOP-lowering drugs. The cumulative probability of requiring IOP-lowering drops was 21.8% at 12 months and 46.2% at 24 months in the overall cohort, with no significant differences between naïve and previously treated eyes. Glaucoma surgery was only required in 1 case (0.49%), that had pre-existing glaucoma and was already on IOP lowering medications prior to first injection. The effect of repeat IDI injections on IOP was also evaluated, and no significant differences were observed in the cumulative probability of any IOP level 6 months after the first, second or third injection.

## Discussion

This study reports better visual outcomes and reduced treatment burden in naïve DME eyes compared to previously treated DME eyes, based on a large cohort of DME patients treated with the IDI in routine clinical care. The results of this study support the use of the IDI in early DME stages in patients who do not qualify for anti-VEGF therapy and provide further evidence of better IDI response when used as first-line therapy compared to its use as second-line therapy, after previous failed intravitreal treatments.

Our real-world cohort of DME patients presented different demographics and baseline clinical characteristics from those reported in clinical trials, overall with a worse mean baseline VA [9, 10, 13, 18, 19] and CRT [9, 10, 18, 19]. In our series, a significant number of study eyes were treated in routine clinical care with VA levels that fall outside the inclusion criteria used in the MEAD trial (33.5% of the study cohort, 6% with VA better than 0.4 logMAR and 27.5% with VA worse than 1.0 logMAR, as presented in Fig. 1). This data is of particular relevance, as reflects more closely the situation in which such a therapy is to be employed in routine clinical care: patients that require a therapeutic option even though they might not conform to the ideal profile, either in terms of specific disease related parameters, prior treatment failure or co-morbidities. In case of our cohort of previously treated eyes, their basal clinical characteristics are generally worse than those reported previously in real-world studies, particularly with lower baseline VA [13, 14, 20–24] and greater CRT [20, 25]. Moreover, more eyes had received prior treatment, particularly with anti-VEGF and/or IVTA, than the study cohorts in these previous series [14, 20, 22, 24–26].

The overall results presented here are somehow comparable with those from previous smaller cohorts. Indeed, previous assessments of the effect of IDI on refractory and treatment-naive patients showed similar decreases in CRT and improvement in VA in both refractory and treatment-naive groups. Other real-life studies have also shown similar improvements in VA and CRT without serious adverse events, mainly in smaller series over shorter follow-up periods [27–32]. In our population of DME patients, IDI considerably improved the VA relative to baseline in both treatment-naive and previously treated patients, suggesting that IDI therapy offers benefits to both types of patient. Nevertheless, naive eyes maintained a better mean VA than previously treated eyes at all time points studied. A particularly significant improvement was observed in naïve patients during the first 3 months relative to previously treated eyes, suggesting they respond better in terms of VA, consistent with earlier data [13, 14]. Moreover, in our series the percentage of eyes with good VA levels was consistently higher in naïve eyes vs previously treated eyes at all study timepoints, as graphically presented in Fig. 1. Regarding anatomical changes, IDI treatment improves the CRT at all time points during the follow-up, both in treatment-naïve and previously treated eyes, with no significant differences between these subgroups. This discrepancy between functional and anatomical outcomes in DME has extensively been reported in previous studies, that suggest that retinal thinning may also be related with outer retinal layers atrophy preventing visual improvement, more common in chronic DME eyes [33].

The need for frequent injections in DME represents a considerable burden for patients, especially with antiVEGF drugs [34–36]. In different studies, fewer IDI injections have been shown to be necessary to achieve similar visual and anatomical outcomes in DME patients [10, 11, 37], although the loss of vision mainly due to cataract must be controlled, which may be more common when IDIs are used. Further head-to-head trials will be needed to compare the efficacy of IDI and anti-VEGF therapy based on the patients’ clinical characteristics at baseline and their prior treatments, tailoring the treatment choice individually in a case-by-case basis. Towards this personalized medicine approach, several attempts have been recently reported to shed some light on predictive biomarkers for IDI response, based in retinal imaging (i.e. OCT) or aqueous samples [38–41]. Likewise, recent studies have been directed to identify those patients who don’t respond to anti-VEGF treatment, [42] as well as to determine the synergistic and beneficial effect of IDIs in combination with other treatments [43, 44].

Significantly fewer IDIs were administered to treatment-naïve eyes than to previously treated eyes and indeed, many more treatment-naïve eyes received just 1 injection than previously treated eyes. However, when additional implants were required, the time to reinjection did not differ significantly between the two groups. This is an important point, as suggest that reinjections were timely performed in both groups when required. Such differences have not always been detected when this parameter has been compared between naïve and non-naïve eyes [14, 20]. Our results raise the interesting hypothesis that early treatment may reduce the treatment burden associated to IDI therapy in naïve DME, beyond the benefit already reported for refractory DME eyes when used as second line therapy. It is well recognized that managing DME with IDIs is generally associated with a need for fewer injections than anti-VEGF therapies, as well as longer periods between the need for treatment. The benefits to be gained from this need for fewer injections have already been recognised in the EURETINA guidelines for DME, whereby IDI use is recommended as a first-line therapy only in specific subgroups of patients. If confirmed in future studies, this finding may offer an additional reason to support the use of IDI as first-line therapy in a wider spectrum of DME eyes.

As found elsewhere, IDI therapy was well tolerated by DME patients. The main adverse event was high IOP but that could typically be managed with medication. Indeed, while nearly a third of patients developed a high IOP, it was controlled with topical antihypertensive drugs. There is no evidence of a previously cumulative effect of multiple injections on increased IOP, irrespective of pre-existing glaucoma or ocular hypertension (OHT) [17, 45]. However, the probability of taking IOP-lowering medication increased considerably from 12 months to 24 months, an important consideration when using the IDI in routine clinical care [46].

Our study has some limitations, such as the retrospective design of the study. In addition, a detailed comparison of the data obtained with that from previous clinical trials and other retrospective studies is complicated by the fact that they involve cohorts with different characteristics at baseline (Table 4). This is particularly the case of DME severity and the inclusion/exclusion criteria, and notably in terms of the baseline VA that differs substantially between trials, as well as the type and length of the prior treatments, and the intervals between the repeated IDI injections. Despite the absence of detailed comparisons, the results of the present study in a clinical setting are somehow comparable to the data from clinical trials. Furthermore, as the data is obtained from a cohort of patients not included in a clinical trial it better reflects patients seen in clinical practice, both treatment-naive and previously treated patients with DME. Therefore, similar outcomes can be expected of IDI treatment at other clinical centres for DME patients, as reported by previous smaller cohorts. Moreover, it is likely that the patients included in this study are patients that might otherwise not be recommended for such therapy as they do not conform to the criteria employed in clinical trials. It should also be noted that some of the patients considered to be naïve to treatment in this study had received prior laser therapy but not any prior intravitreal therapy. This may contribute to explain differences in treatment response and disease progression (and/or adverse events) between this cohort of naïve patients and those studied elsewhere. Finally, two different OCT machines from two different manufacturers were employed in this study (one in each participating center), but CRT measurements were not adjusted for inter-device differences and this data should be interpreted with caution, especially when comparing these results with other clinical practice studies and trial outcomes.

**Table 4:** Comparison of dexamethasone intravitreal implant clinical trials and real-life studies of in diabetic macular edema (series ≥30 eyes)

Study	Indication	Duration (months)	N	Baseline VA (letters)	Baseline CRT (μm)	Baseline %laser	Baseline %anti-VEGF	Baseline %IVTA	Final VA, (letters)	Final CRT (μm)
MEAD Boyer DS et al., 2014 [9]	DME	36	351	56.1	463.0	65.8%	7.1%	16.5%	58.6	351.4
MEAD-Treated Augustin AJ et al., 2015 [19]	DME-Treated	36	247	55.2	478	93.5%	25%	23.5%	58.4	352
CHAMPLAIN Boyer DS et al., 2011 [18]	DME-PPV	6	55	54.5	403.4	69.6%	48.2%	57.1%	57.5	364.5
BEVORDEX Gillies MC et al., 2014 [10]	DME	24	88	55.5	474.3	–	–	–	62.4	287.3 (*)
Escobar-Barranco et al., 2015 [13]	DME-Naive	6	40	59.6	568	0%	–	–	71.1	323
	DME-Treated	6	36	51.3	600	100%	–	–	59.0	281
Dutra et al., 2014 [21]	DME-Treated	6	58	52*	543.24	74.1%	75.9%	67.2%	58.5*	420.16
Totan et al., 2016 [22]	DME-Treated	6	30	57*	517	56.7%	100%	–	64*	411
Bansal et al., 2016 [23]	DME	14.53	52	44*	514.2	100%	67.2%	–	51*	419.9 (6 months)
Bonnin et al., 2015 [24]	DME	4	39	51.5*	559	44%	49%	36%	81.5*	477
Guigou et al., 2015 [26]	DME treated and naive	6	78	53.9	537.6	42.3%	52.6%	17.9%	60.1	384.6
CHROME Lam et al., 2015 [25]	DME (subgroup analysis)	36	34	55*	450.4	55.9%	55.9	38.2	53 (6 months)	259.5
RELDEX Malclès et al., 201 7[20]	DME naive and treated	36	128	50.5	450	16.4%	70.3%	15.6%	60.6	280
IRGREL-DEX Iglicki et al., 2019 [14]	DME naive and treated	24	130	55*	575	15%	7.4%	–	65.5*	294.4**
*This study:* *Zarranz-Ventura* et al. *2020* [6]	***DME-All***	***24***	***203***	***39***	***498.7***	***75.8%***	***53.6%***	***25.6%***	***45***	***402.6***
	***Naive***	***24***	***67***	***42.5***	***482.1***	***56.7%***	***0%***	***0%***	***47***	***340.5***
	***Previously treated***	***24***	***136***	***37.5***	506.5***	***85.2%***	***80.1%***	***38.2%***	***44***	427.4***

DME Diabetic macular edema, *VA* Visual acuity, *CRT* Central retinal thickness, *VEGF* Vascular endothelial growth factor, *IVTA* Intravitreal triamcinolone acetonide. *Logarithm of the minimum angle of resolution values converted into ETDRS letters.** Calculated from publication data. *** CRT measurements obtained with 2 different OCT machines, unadjusted for inter-device differences

## Conclusions

In summary, IDI treatment for DME in our cohort produced favourable outcomes in both naïve and previously treated eyes. These results support the use of IDIs to treat patients with DME in everyday clinical practice, in patients that don’t conform to the strict criteria employed in clinical trials. In addition, the data presented here highlight the need for prospective studies to assess the additional benefits of using IDI over anti-VEGF therapies, and in particular, of the use of such implants in naïve DME patients should this additional effect in reducing the treatment burden when compared to refractory DME patients is confirmed in future studies. Such studies may lead to the extension of the EURETINA guidelines that currently limit the recommendation of IDI as a first-line therapy to only a few specific circumstances to a wider spectrum of DME eyes.

## Data Availability

The datasets used and/or analyzed during the current study are available from the corresponding author on reasonable request.

## Abbreviations

CF: Counting fingers
CRT: Central retinal thickness
DME: Diabetic macular edema
ETDRS: Early treatment diabetic retinopathy study
HM: Hand movement
IDI: Intravitreal dexamethasone implant
IOP: Intraocular pressure
IQR: Interquartile range
IRB: Institutional review board
IVTA: Intravitreal triamcinolone
KM: Kaplan-Meier
LogMAR: logarithm of the mínimum angle of resolution
OCT: Optical coherence tomography
PRP: Panretinal photocoagulation
PT: Previously treated
SD: Standard deviation
VEGF: Vascular endothelial growth factor
VA: Visual acuity
